# Virtual Therapy Planning of Aortic Valve Replacement for Preventing Patient-Prosthesis Mismatch

**DOI:** 10.3390/bioengineering12040328

**Published:** 2025-03-21

**Authors:** Marie Schafstedde, Florian Hellmeier, Jackie Grünert, Bianca Materne, Titus Kuehne, Leonid Goubergrits, Sarah Nordmeyer

**Affiliations:** 1 Department of Congenital Heart Disease—Pediatric Cardiology, German Heart Center Charité, 13353 Berlin, Germany; 2 Institute of Computer-Assisted Cardiovascular Medicine, German Heart Center Charité, 13353 Berlin, Germany; 3 Berlin Institute of Health (BIH), 10178 Berlin, Germany; 4 Deutsches Zentrum für Herz-Kreislauf-Forschung, German Centre for Cardiovascular Research, Partner Site Berlin, 10785 Berlin, Germany; 5 Institute of Biometry and Statistical Epidemiology, Charité Universitätsmedizin Berlin, 10117 Berlin, Germany; 6 Einstein Center Digital Future, 10117 Berlin, Germany; 7 Division of Pediatric Radiology, Department of Radiology, University Hospital Tübingen, 72076 Tübingen, Germany

**Keywords:** aortic valve replacement, virtual therapy planning, CFD, 4D flow MRI, stress simulation

## Abstract

Background: Recent studies suggest that any degree of patient-prosthesis mismatch (PPM) increases morbidity and mortality after surgical aortic valve replacement (SAVR). We used computational fluid dynamics simulations to test the influence of prosthesis size and physical activity after SAVR. Methods: In 10 patients with aortic valve stenosis, virtual SAVR was performed. Left ventricular outflow tract stroke volume and flow direction information (4D Flow) were used, and an increase in stroke volume of 25% was chosen for simulating physical activity. Pressure gradients (DP max) across the aortic valve and blood flow profiles in the ascending aorta were calculated and predicted for three different valve sizes at rest and under stress in every patient. Results: Gradients across the aortic valve were significantly lower using larger valves; however, they were not normalized after SAVR (DP max [mmHg] norm/smaller/reference/larger valve = 6/14/12/9 mmHg, <0.01 compared to norm). Physical activity simulation increased DP max in all patients and across all valve sizes (DP max [mmHg] rest versus stress for the smaller/reference/larger valve = 14 vs. 23, 12 vs. 18, 9 vs. 14). Blood flow profiles did not normalize after SAVR and remained unaffected by physical activity. Gradients differed between mild and moderate stenosis between different therapy options and even showed moderate to severe stenosis under simulated physical activity. Conclusions: Prosthesis size and physical activity simulation have a significant influence on gradients across the aortic valve. Virtual therapy planning using patient-specific data might help to improve outcomes after SAVR in the future.

## 1. Introduction

Patient-prosthesis mismatch (PPM) is a well-known postoperative problem after surgical aortic valve replacement [1,2,3,4,5]. PPM occurs when the effective orifice area (EOA) of the implanted prosthetic valve is too small relative to the patient’s body size. An indexed EOA of ≤0.85 cm^2^/m^2^ is typically considered the threshold for PPM in the aortic position, with values between 0.65 and 0.85 cm^2^/m^2^ classified as moderate PPM and those <0.65 cm^2^/m^2^ as severe PPM. Severe PPM has been linked to poorer hemodynamic function, reduced regression of left ventricular hypertrophy, increased cardiac events, and lower survival rates [6,7,8,9]. Recent publications, however, even described that any degree of PPM increases long-term morbidity and mortality after surgical aortic valve replacement (SAVR), especially using biological valve prostheses [8,10].

Either the surgically implanted valves are too small for the individual patient, or valve degeneration occurs, leading to increased gradients across the aortic valve. In the pediatric population, patient growth accompanied by a rise in cardiac output is another factor contributing to the development of PPM and the need for reoperation. Chronic pressure load on the left ventricle, resulting from increased valvular pressure gradients caused by PPM, leads to cardiac hypertrophy and adverse remodeling, which in turn increases morbidity and mortality. This is particularly crucial in young patients with aortic valve disease, as their life expectancy is expected to exceed more than 50 years. Not only pressure gradients across the aortic valve are relevant in aortic valve disease, but also blood flow profiles and their effects on ascending aortic dilation. Pathological blood flow profiles in the ascending aorta are associated with the progression in aortic wall dilation [11,12], aortic aneurysm formation [13], and aortic dissection [14,15] and were shown to even persist after successful SAVR [16,17] with yet unknown clinical long-term effects. Anatomical restrictions, such as a small aortic annulus, often make it difficult to implant a large enough prosthesis to avoid PPM, particularly in children and smaller women. While it is technically possible to enlarge the aortic annulus to accommodate a larger prosthesis, this approach extends the duration of the procedure and increases the risk of complications [18].

Preventive strategies were outlined in a consensus document, in which the task force recommended that manufacturers use standardized valve charts to present essential information on surgical heart valve characteristics, including physical dimensions, implant position, and hemodynamic performance of the prosthetic valve. However, the accuracy of predicting severe PPM using these charts has been reported to be as low as 59% [19,20].

Individual prediction of hemodynamic outcome after specific therapies could help to improve decision-making if anulus enlargement, for example, should be performed in order to prevent PPM and improve long-term outcome, especially in young patients with a physically active lifestyle. The idea of the study was to evaluate whether the implantation of commercially available valves that are one size larger and one size smaller than the valve actually implanted would have a significant impact on the aortic valve gradient and flow profiles in the ascending aorta. Additionally, we sought to systematically assess the effect of an increase in cardiac output, which could simulate physical activity or patient growth.

In recent decades, computational simulations have increasingly been used to predict postoperative hemodynamic outcomes, with the goal of enhancing decision support, therapy planning, and outcomes across various pathologies [21,22,23]. Individualized preoperative planning, based on patient-specific anatomy and hemodynamic data, is not yet standard practice. However, research has demonstrated that predicting hemodynamic outcomes after SAVR is possible and that different therapies result in varying hemodynamic outcomes in the same patient anatomy [23]. Computational simulations are primarily used to assess resting conditions. However, patients—particularly young adults and children—are more physically active and continue to grow, both of which result in an increase in cardiac output. This increase in volume across the aortic valve raises the pressure gradient, thereby increasing the pressure load on the left ventricle.

The aim of the present study was to use computational simulations to assess the hemodynamic impact of (1) different aortic valve prosthesis sizes and (2) increased amounts of cardiac output simulating physical stress and/or patient growth. The ultimate goal is to achieve the lowest possible gradient across the aortic valve both at rest and during physical activity, as well as to normalize aortic flow profiles for each individual patient.

## 2. Methods

### 2.1. Study Design and Patient Cohort

A total of 10 patients with severe aortic valve stenosis and indication for aortic valve replacement (AVR) were included in the present study. All patients received a Carpentier Edwards Perimount Magna Ease aortic valve prosthesis (Edwards Lifesciences, Irvine, CA, USA). Patient characteristics at baseline, clinical pre- and post-surgery parameters, and the data used for simulation are depicted in Table 1.

All imaging and clinical data used in this study were acquired during the SMART project (ClinicalTrials.gov identifier NCT03172338, German Heart Center Berlin, ethics committee approval ID EA2/133/14, 10 December 2015). Written informed consent was obtained from all patients.

All patient-specific values were compared against a healthy control group. The study cohort has been investigated in previous studies [24,25,26]. Included were 36 female and male volunteers between 50 and 80 years of age, belonging to a random sample of the general population of the city of Freiburg. An exhaustive description of the study cohort is provided in a previous publication by Harloff et al. (2018) [25]. Blood flow parameters were placed in the context of recently published standard values (expressed in percentiles) [27].

### 2.2. Magnetic Resonance Imaging Acquisition

Magnetic Resonance Imaging (MRI) data from 10 patients who underwent surgical AVR at the German Heart Center in Berlin were used. The detailed protocol was previously published [28].

Briefly, all 10 patients were examined by MRI pre- and postoperatively. Cardiac MRI examinations were performed using a 1.5 Tesla MR system (Achieva; Philips Medical Systems, Best, The Netherlands). Balanced 3D steady-state-free-precession (SSFP) imaging during end-diastole (3 signal averages, navigator gated, ECG triggered) was used for reconstruction of the patient-specific anatomy with a typical reconstructed voxel size of 0.80 × 0.80 × 2.00 mm. Four-dimensional flow MRI data were used for flow measurements as a boundary condition for the virtual treatment simulations. The reconstructed voxel size was 2.80 × 2.25 × 2.25 mm with a temporal resolution of 25 phases per cardiac cycle.

All relevant steps for the virtual intervention, including the segmentation process using ZIBAmira (version 2015.28), Zuse Institute Berlin, Berlin, Germany), were previously described in detail [28].

### 2.3. Data Processing Before Virtual Intervention

Anatomical structures, including the left ventricular outflow tract, thoracic aorta, and major arteries, were manually segmented using ZIBAmira (version 2024.07). Additionally, the native aortic valve, coronary artery ostia, and mitral valve leaflets were segmented for orientation during virtual intervention. Geometries of the Perimount Magna Ease prosthesis, including its frame and leaflets, were kindly provided by Claudio Capelli (Institute of Cardiovascular Science, University College London, UK) and used to create a 3D model, scaled to various sizes. Virtual interventions involved positioning the prosthesis model within the preoperative vessel geometry, intersecting the two, and cleaning the resulting surface geometry using STARCCM+ and MeshLab for topological accuracy. A detailed description of the workflow is presented in a previous publication [28].

### 2.4. CFD

CFD simulations were performed using STAR-CCM+ (version 16.06.008, Siemens Digital Industries Software, Plano, TX, USA), which discretizes the governing equations of flow using the finite volume method. A base mesh size of 0.5 mm was chosen, based on a convergence analysis from a previous study [28], with computational mesh sizes varying between 2.6 and 5.1 million cells, depending on the patient. Blood was modeled as an incompressible liquid (ρ = 1050 kg/m^3^), and a modified Carreau–Yasuda model was used for the viscosity (zero shear viscosity μ0 = 0.16 Pa·s, infinite shear viscosity μ∞ = 0.0035 Pa·s, power constant *n* = 0.2128, transition parameter *a* = 0.64, relaxation parameter λ = 8.2 s). Peak systolic steady boundary conditions were set at the in- and outlets while the simulations were performed using a URANS approach due to unsteady flow effects on the valve prostheses’ leaflets. Turbulence was modeled using a shear stress transport k-ω model. Four-dimensional flow MRI-derived flow rates from pre-surgery imaging were prescribed at the inlets using patient-specific velocity profiles. As described below, six simulations, using two different inlet flow rates (reference flow rate and +25% higher) and three valve prosthesis sizes (actually implanted size, one size smaller, one size larger) were performed for each patient. Flow was distributed across the outlets using a combination of 4D flow MRI measurements for the descending aorta and Murray’s law for the branching vessels of the aortic arch. Additionally, flow through the brachiocephalic trunk was assumed to be equal to the combined flow through the left common carotid and left subclavian arteries.

The virtual valve implantation was performed following a protocol that was recently published [28]. Briefly summarized, the virtual interventions involved placing a 3D model of an opened Perimount Magna Ease prosthesis into the preoperative vessel geometry. This was followed by intersecting the prosthesis and vessel geometries and refining the resulting structure using STAR-CCM+ and MeshLab (version 2016.12, Visual Computing Lab, Istituto di Scienza e Tecnologie dell’Informazione, Pisa, Italy) to create a manifold and topologically accurate surface geometry.

Due to the limited availability of clinical data, a simplified computational fluid dynamics (CFD) approach was utilized. Simulations were conducted for peak systolic flow rates, defined as the maximum flow rates occurring during the ejection phase of the cardiac cycle, when the valve-induced transvalvular pressure gradient is expected to be most pronounced. In this modeling framework, both the vessel wall and valve leaflets were assumed to be rigid.

The ability of the steady approach used in this study to accurately assess peak-systolic hemodynamics was investigated in a set of our earlier studies and confirmed by a comparison with 4D flow MRI measurements in patients (flow patterns and maximal valve velocities), with catheter-measured pressure gradients in stenosed aortic valve or by a comparison of pressures and wall shear stresses between steady and unsteady simulations, which used rigid wall assumptions [29,30].

### 2.5. Stress Model

To model the relative increase in maximal flow rate induced by high-intensity dynamic exercise in AS patients, data from the literature were used. Otto et al. directly measured maximal flow rate using echocardiography in 28 study subjects with AS immediately post Bruce protocol exercise [31]. Del Villar et al. measured Doppler-derived stroke volume, systolic ejection period, Doppler-derived aortic valve area, and peak transvalvular velocity during symptom-limited bicycle-exercise testing of 20 aortic stenosis (AS) patients, which can be used to estimate maximal flow rate increase [32]. Based on these studies, we modeled the relative increase in maximal flow rate at 25%.

### 2.6. Calculation of Aortic Blood Flow Parameters

Blood flow parameters quantifying aortic hemodynamics that are analyzed in this study include the normalized flow displacement (NFD), the degree of wall parallelism (WPD), and the flow angle. All quantified blood flow profiles were compared to recently defined normal values, expressed in percentiles, based on the aforementioned study [27].

#### 2.6.1. Normalized Flow Displacement (NFD)

**Figure 1:**
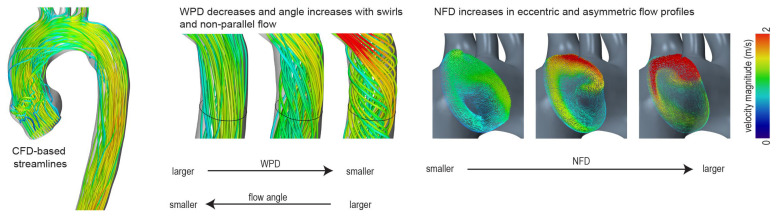
Quantification of blood flow profiles—schematic illustration. WPD—degree of wall parallelism; NFD—normalized flow displacement.

The NFD was initially introduced by Sigovan et al. and describes the distance between the center of the lumen and the aortic forward flow, normalized to the diameter of the aortic cross-section [33]. An absolutely central flow results in an NFD of 0, whereas the NFD increases if the forward flow is shifted to the aortic vessel wall. A schematic illustration is shown in Figure 1.

#### 2.6.2. The Degree of Wall Parallelism (WPD)

The WPD is defined as the average ratio of the through-plane velocity magnitude and the sums of through-plane and in-plane velocity magnitude [27]. A WPD of 0 indicates that all velocity vectors are oriented in-plane. If the flow is perfectly parallel to the centerline orientation, the value is 1. A schematic illustration is shown in Figure 1.

#### 2.6.3. Flow Angle

The angle is defined as the average angular deviation of each measured velocity vector and the normal direction of the evaluated cross-section. It is a parameter that was, among others, used by Entezari et al. and Mahadevia et al. to assess pathological flow profiles in the ascending aorta [34,35].

### 2.7. Statistical Analysis

Patient characteristics, such as demographic information, volumetric measurements, and clinical data, are summarized descriptively for each patient (Table 1). More details can be found in previous studies [16]. Median values of DP max, V max, NFD, WPD, and flow angle are presented according to the valve size (Table 2) and the physical activity level (Table 3 and compared to corresponding data from a healthy control cohort. Additionally, key hemodynamic parameters, including the pressure gradient across the aortic valve (DP max) and blood flow parameters (NFD) stratified by valve size and physical activity, are presented at the individual patient level in Table 4.

The primary hypothesis of this study was that hemodynamic parameters and blood flow profiles vary according to prosthesis size and physical activity following SAVR. The Wilcoxon-signed-rank (WSR) test was used for paired comparisons between different prosthesis sizes and varying levels of physical activity. Additionally, the Wilcoxon-Mann–Whitney (WMW) test was used for comparison with the independent healthy control group. Rank-based statistical methods were selected due to the limited sample size (*n* = 10 patients). For each test, the test statistic, the *p*-value and the relative effect *p_rel_* as a measure of effect size were reported. This value represents the probability that a randomly selected value from sample 2 is higher than a randomly selected value from sample 1. The interpretation is as follows:

➢ If *p_rel_* = 0.5: Both samples contain equally large values in terms of ranks. No difference between the groups can be detected.
➢ If *p_rel_* < 0.5: sample 1 tends to have higher values than sample 2 (the closer *p_rel_* is to 0, the stronger the effect).
➢ If *p_rel_* > 0.5: sample 2 tends to have higher values than sample 1 (the closer *p_rel_* is to 1, the stronger the effect).

To account for multiple testing, *p*-values were adjusted using the Benjamini–Hochberg correction. We specifically opted for this correction method in order to control the false discovery rate, balancing the risk of false positives while maintaining higher statistical power compared to more conservative methods like Bonferroni. A *p*-value of <0.05 was considered statistically significant.

All statistical analyses were exploratory in nature and conducted using the R statistical software and additional packages (version 2023.06.1 Build 524).

## 3. Results

### 3.1. Influence of Aortic Valve Size on Pressure Load for the Left Ventricle and Blood Flow Profiles in the Ascending Aorta

According to current clinical guidelines [36], the normalization of velocities and gradients across the aortic valve could not be achieved after SAVR. Choosing a smaller valve rendered even higher maximal velocities and gradients across the aortic valve, whereas choosing a larger valve resulted in lower values than the reference valve. However, neither led to a normalized gradient across the aortic valve.

The influence of different valve sizes on pressure gradients and blood flow profiles in the ascending aorta as well as their difference to normal values are depicted in Table 2 and Table S1 of the Supplementary Materials.

Blood flow profiles in the ascending aorta only featured small changes after implanting a smaller or larger valve. The degree of wall parallelism (WPD) was significantly lower (WMW, U = 360.0, *p* < 0.01), and the normalized flow displacement (NFD) was significantly higher (WMW, U = 15.0, *p* < 0.01 for the smaller; U = 11.0, *p* < 0.01 for the reference; and U = 15.5, *p* < 0.01 for the larger valve, respectively) when compared to a healthy reference cohort. The flow angle showed significantly higher values compared to a healthy control group (WMW, U = 12.0, *p* < 0.01 for the smaller; U = 41.0, *p* < 0.01 for the reference; and U = 44.5, *p* < 0.01 for the larger valve, respectively). There were no significant differences in blood flow profiles between the valve sizes; however, there were tendencies towards normalization in larger valves. While the flow angle showed a tendency to decrease when a larger prosthetic valve was inserted, the WPD slightly increased when a larger aortic valve was virtually inserted. The NFD did not change depending on the prosthetic valve size (Table 2).

The individual values for key hemodynamic and blood flow parameters, including DP max and NFD, for all 10 patients are presented in Table 4. Additional parameters analyzed in this study, such as Vmax, WPD, and angle, are detailed in Table S1 of the Supplementary Materials.

### 3.2. Influence of Physical Stress Simulation on Pressure Load for the Left Ventricle and Blood Flow Profiles in the Ascending Aorta

Physical stress simulation rendered significantly higher velocities (WSR, W = 55.0, *p* < 0.01 for all valve sizes) and higher gradients (WSR, W = 81.5, *p* = 0.03 for the smaller; W = 78.5, *p* < 0.05 for the reference and W = 77.0, *p* = 0.06 for the larger valve, respectively) across the aortic valves compared to the simulations at rest (Table 3). The smaller the inserted valve, the higher the gradient across the aortic valve, both at rest and under stress, and the higher the mean increase in gradients between rest and stress (DP max rest versus stress: 5.4/5.6/9.0 mmHg for the larger/reference/smaller valve sizes). Note that an increase in DP max due to stress is about two or three times higher than an increase in DP max due to a smaller valve size (3 mmHg difference between reference and larger valve as well as 2 mmHg difference between smaller and reference valves, Table 2).

Blood flow profiles did not change significantly between simulations at rest and at physical stress (Table 3 and Table 4).

### 3.3. Individual Differences Reveal the Need for a Personalized Approach

**Figure 2:**
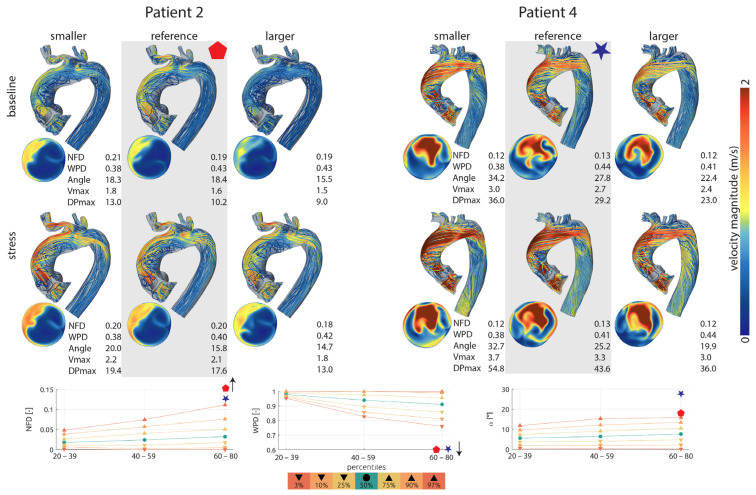
(**Top**): Blood flow profiles, velocity, and pressure gradients under varying valve sizes and levels of physical stress for two exemplary patients. Simulation data for the reference valve is highlighted in gray. (**Bottom**)*:* Blood flow profiles resulting from the reference valve, displayed in percentiles and compared to a healthy control group. NFD—normalized flow displacement; WPD—degree of wall parallelism; angle (alpha)—flow angle; Vmax—maximum velocity; DP max—maximum pressure gradient.

There were noteworthy interindividual differences. One patient almost reached normal values (patient 6) for gradient across the aortic valve and flow profiles, whereas patient 4, for example, showed the highest gradient across the aortic valve after SAVR, mild stenosis at rest, and moderate stenosis at physical stress (Table 4). Figure 2 shows blood flow profiles, velocity, and pressure gradients under varying valve sizes and levels of physical stress for two exemplary patients. In Patient 2, the velocity and pressure gradient of the virtually implanted valve remained low both at rest and under physical stress, whereas in Patient 4, they were significantly higher, as mentioned above. However, the blood flow profiles did not normalize, remaining above the 97th percentile for NFD and flow angle or below the 3rd percentile for WPD, respectively (Figure 2).

Although not completely normalized, none of the cases showed severe aortic valve stenosis with maximal velocities above 4 m/s at rest or at physical stress. At rest, one case in the reference valve group and one case in the smaller than reference group showed velocity values indicating mild stenosis; all the other cases showed lower velocity values than 2.5 m/s (Table S1 of the Supplementary Materials). At simulated stress, three cases in the reference valve group and the smaller than reference valve group showed velocities implying mild stenosis and one case in each group showed moderate stenosis. In the larger than reference group, there was one case of moderate stenosis. All other cases showed lower values than 2.5 m/s (Table S1 of the Supplementary Materials).

## 4. Discussion

The aim of the present study was to investigate the individual hemodynamic impact of different valve sizes and simulated physical activity on gradients across the aortic valve and on blood flow profiles in the ascending aorta after SAVR by using patient-specific CFD simulations based on patient-specific anatomical and flow MRI data.

Our study demonstrated that complete hemodynamic normalization after SAVR is rare and that prosthetic valve size significantly affects hemodynamic parameters across the aortic valve, with smaller prostheses leading to an expected increase in blood flow velocities and pressure gradients, while larger valves result in lower velocities and gradients. These hemodynamic effects were further amplified during physical activity. These effects, however, are of different relevance in individual patients. Some patients already show mild-moderate aortic valve stenosis at rest after SAVR, which can be reduced by choosing a larger valve; however, to receive a result with almost normal values at rest and under stress, an implantation of a much larger valve would probably be necessary. Other patients show almost normal values after SAVR with little change between different valve sizes and between rest and stress simulation. These results highlight the importance of performing individual treatment planning procedures with the patient-specific anatomical and hemodynamic information.

Notably, pathological flow profiles in the ascending aorta persisted post-surgical aortic valve replacement, regardless of valve size, and remained unchanged under physical stress conditions. Thus, to normalize flow profiles, the mere change in the valve size seems not to be sufficient. When compared to a healthy control group, none of the prosthetic valve sizes achieved complete normalization of velocities, pressure gradients, or blood flow profiles.

### 4.1. Potential Advantage of Including Physical Activity Simulation into Treatment Planning Procedures

Surgical aortic valve repair is a common treatment for aortic valve disease and includes the implantation of biological or mechanical prosthetic valves, the Ross procedure with removing the diseased aortic valve and replacing it with the patient’s own pulmonary valve (autograft), as well as the reconstruction of the affected aortic valve. While all procedures are generally successful in alleviating valve-related symptoms and improving overall cardiovascular health, PPM remains a recognized issue, increasing left ventricular workload and, over time, the overall risk of arrhythmias, morbidity, and mortality [8,10,37].

Especially for younger patients, for example, patients with a bicuspid aortic valve and reasonable early-in-life indication for SAVR, maintaining normal gradients following valve replacement is crucial. These patients normally do not have any comorbidities like diabetes and coronary heart disease and have a higher general life expectancy compared to the common elderly patient with aortic valve disease. Therefore, the selection of the appropriate valve prosthesis is essential to ensure optimal long-term outcomes and reduce reoperation rates. Using larger prosthetic valves and, if necessary, performing aortic root or annulus enlargement procedures seems to minimize the risk of high gradients [38,39,40]. Since these procedures lengthen the operation and increase the risk of complications, a detailed and patient-specific risk-benefit assessment with an individual and multimodal treatment planning procedure is needed.

Traditionally, PPM has been evaluated under resting conditions [4,40], with limited research exploring changes in hemodynamic parameters during stress [41,42]. However, this study underscores the potential for hemodynamic conditions to worsen under stress, leading some patients with normal gradients at rest to develop high gradients during stress. This phenomenon is particularly relevant for younger patients with an active lifestyle and ongoing physical development, as their increasing stroke volumes and cardiac output over time may exacerbate the effects of a so-called “stress-induced PPM”.

To address these dynamic changes, it is crucial to incorporate preoperative stress simulations into treatment planning. Such simulations could help identify patients at higher risk of worsening hemodynamic conditions under stress, enabling tailored treatment strategies. These strategies might include selecting larger prosthetic valves or considering aortic annulus enlargement to mitigate the risks and optimize long-term outcomes.

These findings highlight the importance of integrating hemodynamic changes under physical stress into routine preoperative clinical assessments and present an approach that could pave the way for establishing defined severity levels for what may be termed “stress-induced PPM” in the future.

### 4.2. Simulation of Blood Flow Profiles and Their Value in the Treatment Planning Procedure

The evaluation of blood flow profiles in the ascending aorta additionally provides valuable clinical information regarding disease severity, risk stratification, and treatment planning [33,34,35]. Abnormal flow patterns can have profound consequences, affecting not only aortic valve function but also the integrity of the aorta, potentially leading to aortic dilatation and an increased risk of aortic dissection or rupture [35,43,44]. Blood flow deviations can lead to disturbed flow patterns within the aorta, potentially contributing to aortic wall stress, endothelial dysfunction, and long-term structural changes [45,46]. In agreement with former studies, our findings revealed that blood flow profiles do not normalize towards values found in the control group after SAVR with a biological valve [16]. Pathological blood flow profiles even persist when choosing a different valve size or increasing stroke volume, which represents an interesting finding that has not yet been described in previous studies.

There may be other factors than the mere size of the aortic valve orifice or cardiac output, which exert a substantial influence on blood flow profiles, such as left ventricular outflow tract, aortic arch geometry, or ventricular contraction patterns, for example. In a former study, it could be shown that performing multiple patient-specific changes in valve size, ascending aorta reduction, and/or angulation of the implanted valve has the potential to normalize ascending aorta blood flow profiles [22].

The consideration of blood flow profiles pre- or postoperatively is not yet part of the routine clinical assessment and follow-up even though its many negative long-term effects are commonly recognized. We showed that the median of all blood flow parameters included in this study (WPD, NFD, flow angle) was significantly different compared to a healthy control group, not only pre- or postoperatively but also in resting and stress conditions. Compared to newly defined percentiles [27], in all except one patient, the blood flow parameters either exceeded the 97th percentile or fell below the 3rd percentile in all patients, respectively. Among the 10 patients included in this study, one patient even presented with NFD values above 0.2 after virtual valve replacement, a threshold that has been associated with a fourfold increased growth rate of the aortic diameter and, consequently, an elevated risk of progression of ascending aortic dilation [11]. This preoperative information might justify investigating other treatment options than originally planned.

### 4.3. The Future Role of Virtual Therapy Planning for Patient-Specific Surgical Aortic Valve Replacement

The integration of computational simulations into individualized therapy planning is increasingly recognized as essential [47,48,49,50]. However, clinical adoption remains limited, likely due to several factors. Computational fluid dynamics (CFD) simulations are inherently complex, necessitating substantial technical expertise and computational resources.

One potential solution to address these technical challenges is the application of machine learning algorithms, which, once developed, can generate results in near real-time. For clinical translation, artificial intelligence-based approaches offer significant promise in enhancing clinical decision-making through non-invasive models. Noteworthy examples include machine learning-based methods for predicting obstructive coronary artery disease using treadmill exercise-induced ECG characteristics, as well as a novel multimodal deep learning model for predicting short-term mortality in patients with acute pulmonary embolism [51,52].

Moreover, established medical guidelines and standard operating procedures, particularly in adult medical care, have been in place for years, yielding favorable short- to mid-term outcomes for patients over 60 [53,54]. In routine clinical practice, decisions regarding valve size selection or the need for procedures like aortic annulus enlargement are typically made intraoperatively by the surgeon. Additionally, many in silico modeling approaches documented in the literature are primarily utilized for transcatheter aortic valve implantation (TAVI) planning, a procedure predominantly performed in elderly patients [36,55]. Consequently, therapy planning for younger patients remains an unmet medical need. In this study, we introduced a novel model-based approach designed to predict hemodynamic changes across various aortic valve prosthesis sizes under both resting and stress conditions. This method holds promise for enhancing therapy planning, especially for younger patients who experience increases in cardiac output and ventricular pressure load over time due to growth and an active lifestyle.

## 5. Limitations

This study has several limitations. Firstly, due to the relatively small sample size and a median patient age of 64 years, the findings may not be generalizable to broader populations, and the statistical analyses are inherently descriptive and exploratory in nature.

Secondly, the exclusive inclusion of patients with biological valve prostheses limits the applicability of the results to other types of valve prostheses, suggesting a need for further research in this area.

Thirdly, physical activity was uniformly simulated by increasing stroke volume by 25% across all patients, which does not account for individual variations in physical stress response. This standardized approach may not accurately reflect the diverse hemodynamic responses observed in clinical settings.

Finally, a simplified CFD model with rigid wall assumptions was applied.

## 6. Conclusions

Our study demonstrates that aortic valve size and physical stress simulations significantly influence postoperative gradients across the aortic valve following surgical aortic valve replacement (SAVR) with a biological prosthesis in patient-specific geometrical models. Notably, gradients across the aortic valve and blood flow profiles did not normalize post-SAVR, regardless of valve size or physical stress conditions, however, showing patient-specific differences. Predicting individual postoperative hemodynamic outcomes and testing different treatment options may offer potential advantages for clinical decision-making in the future.

The methodology presented herein shows promise in identifying patients at increased risk of stress-induced patient-prosthesis mismatch, serving as an adjunctive decision support system to enhance individualized patient outcomes. Incorporating this approach into clinical practice may enable healthcare providers to tailor treatment strategies more effectively, potentially reducing postoperative complications and improving overall quality of life.

## Figures and Tables

**Table 1:** Patient characteristics and clinical parameters: pre- and post-surgery.

Patient	Sex [F/M]	Aortic Regurgitation [No/Mild/Moderate]	Height [cm]	Weight [kg]	BSA [m^2^]	Age [Years]	Reference Valve Size [mm]	Peak Systolic Flow Rate [ml/s]	MMi [g/m^2^]	LV-EF [%]	NT-Pro BNP [ng/l]
	Patient Characteristics at Time of Surgery						Data Used for Simulation		Clinical Parameters Post-Surgery		
1	F	no	164	68	1.8	69	23	354	50	69	247
2	M	mild	192	95	2.3	62	27	430	55	53	170
3	M	no	185	98	2.3	72	25	394	64	52	210
4	M	moderate	198	105	2.4	64	23	539	73	57	366
5	M	no	180	81	2.1	60	23	367	71	63	96
6	F	moderate	168	68	1.8	64	23	235	44	54	197
7	M	no	184	100	2.3	70	23	407	46	61	335
8	F	mild	170	70	1.9	57	21	296	47	64	516
9	M	no	169	88	2.1	68	25	412	62	54	250
10	M	no	190	110	2.4	59	25	464	59	54	63
Median			182	95	2.2	64	24	409	55	62	155

F—female; M—male; BSA—body surface area; MMi—indexed muscle mass; LV-EF—left ventricular ejection fraction; NT-pro BNP—N-terminal pro b-type natriuretic peptide. Classification of aortic insufficiency according to regurgitation fraction (RF): no = RF > 10%, mild = RF 10–20%, moderate = RF > 20%.

**Table 2:** The influence of different valve sizes on median pressure gradients across the aortic valve and median blood flow profiles in the ascending aorta as well as their difference to normal values.

Parameters	Healthy Reference Cohort *p*-Value (WMW)	Smaller Valve *p*-Value * (WSR) *p_rel_*	Reference Valve	Larger Valve *p*-Value * (WSR) *p_rel_*
DP max [mmHg]	6 **<0.01 for all valve sizes**	14 (<0.01) * 0.68	12	9 (<0.01) * 0.32
V max [m/s]	1.2 **<0.01 for all valve sizes**	1.9 (<0.01) * 0.68	1.7	1.5 (<0.01) * 0.32
Flow Angle [°]	8 **<0.01 for all valve sizes**	30 (0.11) * 0.58	28	24 (<0.05) * 0.33
NFD	0.03 **<0.01 for all valve sizes**	0.12 (0.59) * 0.52	0.12	0.12 (0.07) * 0.41
WPD	0.75 **<0.01 for all valve sizes**	0.41 (0.19) * 0.40	0.44	0.48 (0.07) * 0.65

DP max—maximum pressure gradient; V max—maximum velocity; NFD—normalized flow displacement; WPD—degree of wall parallelism; *p*-value (based on the Wilcoxon-Mann–Whitney (MWM) test)—in comparison to smaller/reference/larger valves; *p*-value * (based on the Wilcoxon-signed-rank (WSR) test)—in comparison to reference valves; *p_rel_*—relative effect; significant *p*-values are highlighted in bold.

**Table 3:** The influence of different valve sizes on median pressure gradients across the aortic valve and median blood flow profiles in the ascending aorta at rest and during stress.

Parameters	Smaller Valve Rest/Stressed *p*-Value (WSR) *p_rel_*	Reference Valve Rest/Stressed *p*-Value (WSR) *p_rel_*	Larger Valve Rest/Stressed *p*-Value (WSR) *p_rel_*
DP max [mmHg]	14/23 **(0.03)** 0.18	12/17.6 **(0.05)** 0.21	9/14.4 (0.06) 0.23
V max [m/s]	1.9/2.4 **<0.01** 0.18	1.7/2.1 **<0.01** 0.04	1.5/1.9 **<0.01** 0.23
Flow Angle [°]	30/31.2 (0.16) 0.53	28/27.25 (1) 0.50	24/23.9 (0.87) 0.45
NFD	0.12/0.13 (0.62) 0.47	0.12/0.12 (0.76) 0.47	0.12/0.12 (0.28) 0.54
WPD	0.41/0.42 (0.87) 0.49	0.44/0.45 (0.11) 0.54	0.48/0.48 (1) 0.51

DP max—maximum pressure gradient; V max—maximum velocity; NFD—normalized flow displacement; WPD—degree of wall parallelism; WSR—Wilcoxon-signed-rank test; significant *p*-values are highlighted in bold. *p_rel_*—relative effect.

**Table 4:** DP max and NFD values for all 10 patients at rest and under stress.

Patient	Parameters	Smaller Valve Rest/Stressed	Reference Valve Rest/Stressed	Lager Valve Rest/Stressed
1	DP max [mmHg] NFD	14/23 0.12/0.13	12/18 0.12/0.12	9/14 0.10/0.09
2	DP max [mmHg] NFD	13/19 0.21/0.20	10/18 0.19/0.20	9/13 0.19/0.18
3	DP max [mmHg] NFD	19/29 0.14/0.14	16/25 0.12/0.14	14/23 0.13/0.14
4	DP max [mmHg] NFD	36/55 0.12/0.12	29/44 0.13/0.13	23/36 0.12/0.12
5	DP max [mmHg] NFD	14/21 0.12/0.13	10/16 0.10/0.11	8/12 0.09/0.10
6	DP max [mmHg] NFD	8/12 0.04/0.04	7/10 0.05/0.03	6/9 0.04/0.04
7	DP max [mmHg] NFD	21/31 0.12/0.12	16/25 0.12/0.12	13/19 0.12/0.12
8	DP max [mmHg] NFD	14/23 0.08/0.08	10/16 0.08/0.09	8/13 0.07/0.06
9	DP max [mmHg] NFD	14/21 0.14/0.15	12/18 0.14/0.14	9/14 0.13/0.12
10	DP max [mmHg] NFD	21/31 0.13/0.13	16/25 0.13/0.13	14/21 0.12/0.12

DP max—maximum pressure gradient; NFD—normalized flow displacement.

## Data Availability

The original contributions presented in this study are included in the article/Supplementary Materials. Further inquiries can be directed to the corresponding author.

## Supplementary Materials

The following supporting information can be downloaded at: https://www.mdpi.com/article/10.3390/bioengineering12040328/s1, Table S1: DP max V max, NFD, WPD and flow angle values for all 10 patients at rest and under stress.

## Abbreviations

AS	aortic stenosis
CFD	computational fluid dynamics
DP max	maximum pressure gradient
MMi	indexed muscle mass
MRI	Magnetic Resonance Imaging
NFD	normalized flow displacement
PPM	patient-prosthesis mismatch
(S)AVR	(surgical) aortic valve replacement
TAVI	transcatheter aortic valve implantation
V max	maximum velocity
WPD	degree of wall parallelism
