# Supplementary material for: Virtual Therapy Planning of Aortic Valve Replacement for Preventing Patient-Prosthesis Mismatch
Source: Bioengineering (Basel). 2025 Mar 21;12(4):328. doi: 10.3390/bioengineering12040328 (PMC12024315; doi:10.3390/bioengineering12040328)
Supplement: Supplementary file 1 [file bioengineering-12-00328-s001.zip › bioengineering-3507425-supplementary.pdf]

Supplementary Material

Table S1: DP max V max, NFD, WPD and flow angle values for all 10 patients at rest and under stress

Patient	*Parameters*	*Smaller valve rest / stressed*	*Reference valve rest / stressed*	*Lager valve rest / stressed*
1	DP max [mmHg]	14 / 23	12 / 18	9 / 14
	V max [m/s]	1.9 / 2.4	1.7 / 2.1	1.5 / 1.9
	NFD	0.12 / 0.13	0.12 / 0.12	0.10 / 0.09
	WPD	0.36 / 0.35	0.36 / 0.39	0.43 / 0.42
	Flow angle [°]	28 / 30	32 / 31	29 / 30
2	DP max [mmHg]	13 / 19	10 / 18	9 / 13
	V max [m/s]	1.8 / 2.2	1.6 / 2.1	1.5 / 1.8
	NFD	0.21 / 0.20	0.19 / 0.20	0.19 / 0.18
	WPD	0.38 / 0.38	0.43 / 0.40	0.43 / 0.42
	Flow angle [°]	18 / 20	18 / 16	16 / 15
3	DP max [mmHg]	19 / 29	16 / 25	14 / 23
	V max [m/s]	2.2 / 2.7	2.0 / 2.5	1.9 / 2.4
	NFD	0.14 / 0.14	0.12 / 0.14	0.13 / 0.14
	WPD	0.42 / 0.42	0.46 / 0.48	0.55 / 0.54
	Flow angle [°]	32 / 32	28 / 31	27 / 29
4	DP max [mmHg]	36 / 55	29 / 44	23 / 36
	V max [m/s]	3.0 / 3.7	2.7 / 3.3	2.4 / 3.0
	NFD	0.12 / 0.12	0.13 / 0.13	0.12 / 0.12
	WPD	0.38 / 0.38	0.44 / 0.41	0.41 / 0.44
	Flow angle [°]	34 / 33	28 / 25	22 / 20
5	DP max [mmHg]	14 / 21	10 / 16	8 / 12
	V max [m/s]	1.9 / 2.3	1.6 / 2.0	1.4 / 1.7
	NFD	0.12 / 0.13	0.10 / 0.11	0.09 / 0.10
	WPD	0.45 / 0.44	0.47 / 0.50	0.52 / 0.54
	Flow angle [°]	26 / 26	25 / 21	17 / 16
6	DP max [mmHg]	8 / 12	7 / 10	6 / 9
	V max [m/s]	1.4 / 1.7	1.3 / 1.6	1.2 / 1.5
	NFD	0.04 / 0.04	0.05 / 0.03	0.04 / 0.04
	WPD	0.47 / 0.42	0.41 / 0.50	0.50 / 0.51
	Flow angle [°]	13 / 5	5 / 2	6 / 3
7	DP max [mmHg]	21 / 31	16 / 25	13 / 19
	V max [m/s]	2.3 / 2.8	2.0 / 2.5	1.8 / 2.2
	NFD	0.12 / 0.12	0.12 / 0.12	0.12 / 0.12
	WPD	0.41 / 0.42	0.44 / 0.43	0.45 / 0.43
	Flow angle [°]	36 / 36	34 / 34	31 / 32
8	DP max [mmHg]	14 / 23	10 / 16	8 / 13
	V max [m/s]	1.9 /2.4	1.6 / 2.0	1.4 / 1.8
	NFD	0.08 / 0.08	0.08 / 0.09	0.07 / 0.06
	WPD	0.49 / 0.45	0.51 / 0.48	0.50 / 0.50
	Flow angle [°]	33 / 33	33 / 34	26 / 32
9	DP max [mmHg]	14 / 21	12 / 18	9 / 14
	V max [m/s]	1.9 / 2.3	1.7 / 2.1	1.5 / 1.9
	NFD	0.14 / 0.15	0.14 / 0.14	0.13 / 0.12
	WPD	0.53 / 0.49	0.52 / 0.51	0.56 / 0.56
	Flow angle [°]	14 / 13	11 / 11	9 / 8
10	DP max [mmHg]	21 / 31	16 / 25	14 / 21
	V max [m/s]	2.3 / 2.8	2.0 / 2.5	1.9 / 2.3
	NFD	0.13 / 0.13	0.13 / 0.13	0.12 / 0.12
	WPD	0.41 / 0.40	0.41 / 0.41	0.42 / 0.42
	Flow angle [°]	32 / 33	31 / 29	28 / 28
